# Polymorphonuclear Cells Show Features of Dysfunctional Activation During Fatal Sepsis

**DOI:** 10.3389/fimmu.2021.741484

**Published:** 2021-12-13

**Authors:** Marcela Hortová-Kohoutková, Marco De Zuani, Petra Lázničková, Kamila Bendíčková, Ondřej Mrkva, Ivana Andrejčinová, Alexandra Mýtniková, Ondřej Polanský, Kamila Kočí, Veronika Tomášková, Vladimír Šrámek, Martin Helán, Jan Frič

**Affiliations:** ^1^ International Clinical Research Center, St. Anne’s University Hospital, Brno, Czechia; ^2^ Department of Biology, Faculty of Medicine, Masaryk University, Brno, Czechia; ^3^ Department of Anesthesiology and Intensive Care, Faculty of Medicine, Masaryk University, Brno, Czechia; ^4^ Department of Modern Immunotherapy, Institute of Hematology and Blood Transfusion, Prague, Czechia

**Keywords:** sepsis, polymorphonuclears, septic shock, transcriptomics, proteomics, dysfunctionality

## Abstract

Sepsis and septic shock remain leading causes of morbidity and mortality for patients in the intensive care unit. During the early phase, immune cells produce various cytokines leading to prompt activation of the immune system. Polymorphonuclear leukocytes (PMNs) respond to different signals producing inflammatory factors and executing their antimicrobial mechanisms, resulting in the engulfment and elimination of invading pathogens. However, excessive activation caused by various inflammatory signals produced during sepsis progression can lead to the alteration of PMN signaling and subsequent defects in their functionality. Here, we analyzed samples from 34 patients in septic shock, focusing on PMNs gene expression and proteome changes associated with septic shock. We revealed that, compared to those patients who survived longer than five days, PMNs from patients who had fulminant sepsis were characterized by a dysfunctional hyper-activation, show altered metabolism, and recent exit from the cell cycle and signs of cellular lifespan. We believe that this multi-omics approach, although limited, pinpoints the alterations in PMNs’ functionality, which may be rescued by targeted treatments.

## Introduction

Sepsis is a highly heterogeneous, life-threatening organ dysfunction mainly caused by the dysregulated host response to infection. Although the mortality rate seems to be declining, sepsis is responsible for nearly 11 million deaths worldwide every year (1, 2).

Upon infection, immune and endothelial cells activation through their pathogen recognition receptors (PRRs) cause a massive release of inflammatory mediators (3, 4). Making up 40% to 60% of total circulating white blood cells, neutrophils are among the first responders during infection and are known to play a key role in the onset and progression of sepsis (5). These cells are equipped with a wide plethora of innate receptors and antimicrobial molecules and can quickly invade the sites of inflammation where they eradicate pathogens through phagocytosis and the release of neutrophil extracellular traps (NETs) (6). Moreover, neutrophil activation *via* PRRs induces the release of several pro-inflammatory cytokines and chemokines, which, together with the signals coming from the microenvironment and the engagement of adhesion molecules (as selectins and integrins), further increase the migration of circulating neutrophils to the site of infection (7–10). During sepsis, neutrophil numbers in the peripheral blood are increased by a massive release of mature and immature neutrophils from the bone marrow as a result of the signaling from specific pro-inflammatory cytokines (such as TNFα, IL-1β) (8). Furthermore, neutrophils display prolongated lifespan corroborating their role as a key player in sepsis development and resolution (11, 12). Indeed, neutrophils have been extensively studied in the context of sepsis and many studies suggest that most of their functions are impaired during pathology progression (5, 8, 11, 13). However, the majority of the studies only compared the phenotypes of PMNs from sepsis patients with those of healthy donors and thus, making it difficult to detect the specific changes that are associated with the worst outcomes. We have previously shown important roles of monocytes in sepsis onset (4). In this study, we isolated PMNs from the peripheral blood of patients at the onset of septic shock and during its progression, aiming to identify the signature in gene expression or in proteome enabling to predict the severity of sepsis progression. Through transcriptomic and proteomic approaches, we analyzed the phenotypic hallmarks of the PMN pool that are associated with a fulminant death in the Intensive care unit (ICU) or to survival.

## Material And Methods

### Cohort Design

In this prospective study, 34 patients admitted with a diagnosis of septic shock to the Anesthesiology and Resuscitation Unit of the St. Anne’s Hospital in Brno, Czech Republic, were enrolled. Blood samples were obtained within 12 hours (TP1) and 5 days (TP2) after admission to the ICU. Patients with chronic immunosuppression and those who had antibiotic therapy longer than two days were excluded from the study. Cohort details are summarized in **Tables 1A**, **1B**.

**Table 1A:** Clinical characterization of patients (RNA sequencing).

Characteristic		Total	D5+ Survivors	Early deceased	*P* value
**Recruited patients**		11 (100%)	6 (54.5%)	5 (45.5%)	–
**Sex**	Female	4 (100%)	2 (50%)	2 (50%)	–
	Male	7 (100%)	3 (42.9%)	4 (57.1%)	–
**Age, mean (range)**		72.6 (51–89)	72.2 (51–89)	73.0 (66–80)	0.972
**SOFA, mean**		12.2	11.4	12.8	0.735
**Leukocytes, mean (10^3^/μL)**		29.0	17.5	40.4	0.125

SOFA, sequential organ failure assessment.

**Table 1B:** Clinical characterization of patients (Proteome analysis).

Characteristic		Total	D5+ Survivors	Early deceased	*P* value
**Recruited patients**		34 (100%)	27 (79.4%)	7 (20.6%)	–
**Sex**	Female	15 (100%)	12 (80%)	3 (20%)	–
	Male	19 (100%)	15 (78.9%)	4 (21.1%)	–
**Age, mean (range)**		70.6 (49–88)	69.6 (49–88)	74.9 (66–85)	0.217
**SOFA, mean**		11.7	10.9	14.9	**0.011**
**Leukocytes, mean (10^3^/μL)**		19.2	17.9	24.2	0.085

SOFA, sequential organ failure assessment.

Bold value indicates significant P value between tested groups.

Written informed consents were obtained from all enrolled patients and all procedures and protocols were approved by the institutional ethic committee (4G/2018).

### Blood Sample Isolation and Preparation

Blood samples were processed within 2 hours of collection. Polymorphonuclear cells (PMNs) were isolated from peripheral blood after gradient centrifugation using Lymphoprep^®^ (Alere Technologies AS; Oslo, Norway) (density 1.077 g/ml) following the manufacturer’s recommendations, carefully harvesting the top layer of the high-density pellet. The remaining red blood cells were lysed with RBC lysis buffer (BD Bioscience, Franklin Lakes, New Jersey, USA) and clean PMNs were washed twice with phosphate buffered saline (PBS). PMNs were directly lysed in TRI-reagent (Sigma Aldrich, St. Luis, Missouri, USA) and immediately frozen at -80°C. Plasma was collected from the centrifuged samples and immediately frozen and stored at -80°C until use.

### S100A8/S100A9 ELISA

Preparation and measurement of plasma samples were performed with the Human S100A8/S100A9 Heterodimer DuoSet ELISA kit (R&D Systems, Minneapolis, Minnesota, USA) according to the manufacturer’s guidelines. Diluted plasma samples (1:5,000) were incubated overnight. The absorbance was measured at 450nm immediately after the addition of the stop solution.

### Total RNA and Protein Isolation

Total RNA and proteins were isolated from TRI-reagent-lysed samples after the addition of 20% chloroform and centrifugation (12,000 g; 15 min; 4°C). Total RNA was isolated from the aqueous phase using the RNeasy mini kit (QIAGEN, Düsseldorf, Germany) according to the manufacturer’s recommendations. Proteins were isolated from the organic phase by acetone precipitation (Sigma Aldrich, St. Luis, Missouri, USA), followed by three washes with guanidine hydrochloride (Sigma Aldrich, St. Luis, Missouri, USA).

### RNAseq

An Illumina sequencing library was prepared using the NEBNext^®^ Ultra™ II Directional RNA Library Prep Kit for Illumina (New England Biolabs, MA, USA) following the manufacturer’s instruction. 250 ng of total RNA was used as an input into the polyA enrichment module protocol. Enriched samples were fragmented and transcribed into cDNA. Following universal adapter ligation, samples were barcoded using NEB dual indexing primers and pooled equimolarly after picogreen quantitation. The sample pool was sequenced using a Nextseq 550 sequencer (Illumina, USA) and a 75 cycles High output cartridge.

### RNAseq Data Analysis

Raw reads were quality checked, preprocessed, and mapped to the reference genome (Ensembl GRCh38) with gene annotation (Ensembl v94). Mapped reads were counted and summarized to genes. Differentially expressed genes (DEGs) were identified using the DESeq2 v1.30.1 pipeline (14); significant DEGs were defined as those with adjusted p ≤ 0.05 and |logFC| ≥ 1.5.

Gene Ontology (GO) and Gene Set Enrichment Analyses (GSEA) were performed using the R/Bioconductor package *clusterProfiler* v3.18.1 (15). All the analyses were performed in the R (v4.0.3) environment (16).

The immune cell compositions provided in **Supplementary Figure 1** were estimated using quanTIseq and MCPCounter, accessed *via* the R package *immunedeconv* (17–19). The un-normalized read count matrix was used as input for both the analytical tools. The deconvolution was performed in the Conda v4.10.1 environment.

**Figure 1:**
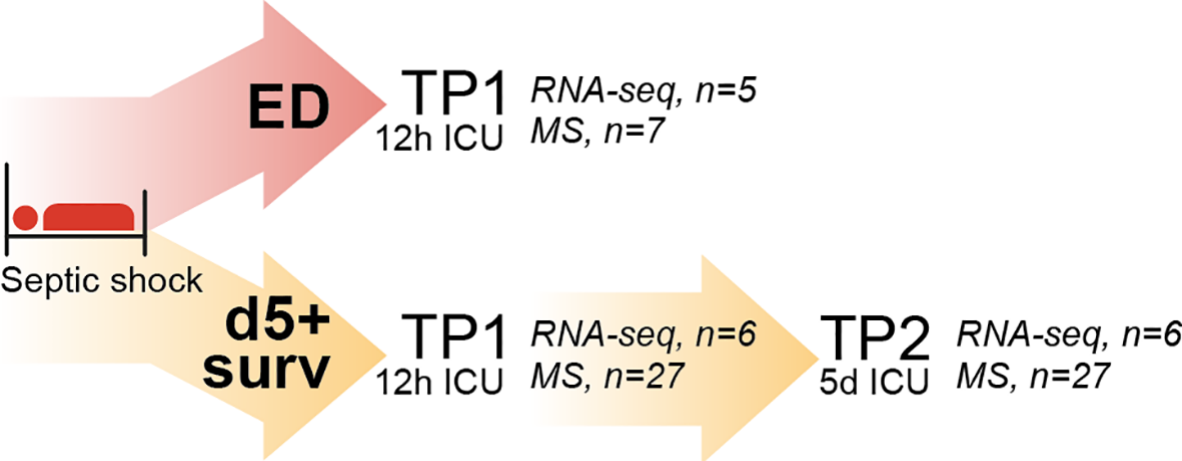
Schematic representation of the experimental design.

The curated gene set used for the cell cycle analysis in **Figure 1** was obtained from a meta-analysis of cell cycle-related genes published by Fischer et al. (available as Table S6 of the original manuscript by Fischer et al.) (20). Genes were selected if present in at least 3 of the 5 studies used for the meta-analysis and assigned to either G1/S or G2/M gene set. The curated gene set is available in **Supplementary Table 1**.

T-cell receptor (TCR) repertoire analysis was performed with MiXCR v3.0.13 and *immunarch* v0.6.5 (21, 22). Unprocessed fastq files were used as input for the MiXCR pipeline. After read alignment, contig assembly, an extension of incomplete sequences, and clonotype assembly, TCR clonotypes were imported in the R environment with *immunarch*, analyzed, and annotated with the VDJdb database (23).

The complete dataset is publicly available for download with the GEO accession number GSE180387.

### Protein Digestion

The protein pellet was dissolved in 300 µl of 8M urea and after centrifugation (2 min; 5,000 g), 250 µl was loaded on 10 kDa MWCO filter (Amicon) and processed by FASP method. Briefly, initial washing of proteins was performed with 8M urea followed by centrifugation for 15 min at 14,000 g. The reduction of the disulfide bonds was performed with 10 mM dithiothreitol for 15 min at room temperature, acetylation was done with 50 mM iodoacetamide for 15 min at room temperature. After 3 washings with 25 mM triethylammonium bicarbonate, trypsin (Promega) was added at a 1:50 ratio and the digestion proceeded overnight at 37°C.

### Mass Spectrometry

LC-MS/MS analysis of tryptic peptides was performed using a Dionex UltiMate 3000 RSLCnano liquid chromatograph connected to micrOTOF-Q II mass spectrometer (Bruker). Samples were separated on C18 Acclaim Pepmap RSLC separation column (25 cm, I.D. 75 µm, particles 2 µm) using a flow rate of 300 nl/min of solvent A (0.1% formic acid) and solvent B (0.1% formic acid in 20/80 H_2_O/ACN (vol/vol)) mixed in 90 min-long linear gradient from 4% to 55% of solvent B. The mass spectrometer was operated at a scanning frequency of 4 Hz and in a data-dependent mode. The five most intensive precursor ions were fragmented using CID fragmentation using an isolation width of 1.2 Th. The collision energy was adjusted between 27 - 48 eV as a function of the m/z value. Dynamic exclusion of fragmented precursor was enabled for 30 s.

### Mass Spectrometry Data Processing and Analysis

Raw LC-MS/MS data were processed by MaxQuant. MS/MS spectra identification was performed using Andromeda search engine using Homo sapiens protein database, 40 ppm, and 0.07 Da as a parent and fragment tolerances (respectively), and oxidation (M) and carbamidomethylation (C) as potential and fixed modifications (respectively). Only proteins passing 5% FDR were kept for further processing. Raw LFQ intensities were log2 transformed. Data were processed using MetaboAnalyst (24). Features with <50% missing values were removed from subsequent analysis. Missing values were replaced by using KNN (feature-wise). The obtained data were analyzed in the R environment using the *Pheatmap* package and in Prism^®^ software. The proteome dataset is publicly available on the jPOST repository (25), with PXID - PXD029219.

### Statistical Analysis

Prism^®^ (GraphPad Software, LLC, Ltd, La Jolla, CA, USA) software and R software were used for statistical analysis. Data were tested for normal distribution and statistical tests were applied as appropriate. Error bars are represented by SD. Statistical tests used are specified in the figure legends. The level of statistical significance was determined: *(*P <*0.05), **(*P <*0.01), and *** (*P <*0.001).

## Results

### Clinical Characterization of the Cohort

We enrolled in total 34 patients to this study, from whom 27 patients survived longer than 5 days after ICU admission (**Figure 1**). Based on the used methodical approach, we divided clinical characterization data into two separate tables. We were not able to perform the sequencing of all patient samples because some of the samples did not meet the technical quality criteria.

### Transcriptome Analysis

#### PMN From Early Deceased Patients Show Signs of Early Hyperactivation

**Figure 2:**
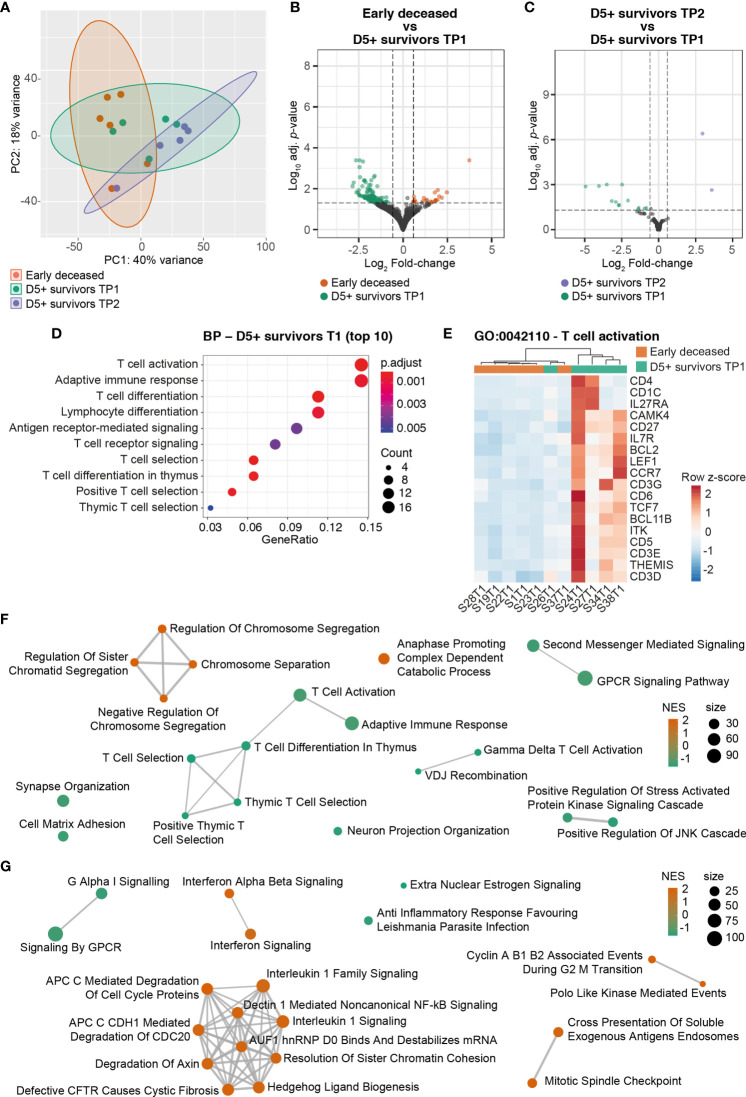
Transcriptional profiling of PMN isolated from sepsis patients **(A)** Principal component analysis showing the overall transcriptomic differences between early deceased patients, D5+ survivors at TP1 and TP2. **(B)** Volcano plot showing the DEGs identified comparing early deceased patients with D5+ survivors at TP1. **(C)** Volcano plot showing the DEGs identified comparing D5+ survivors at TP2 and D5+ survivors at TP1. **(D)** Top 10 results (ordered by *adjusted P* value) of the Gene Ontology analysis performed on the Biological Process (BP) database with the DEGs associated to D5+ survivors at TP1, compared to early deceased patients. **(E)** Heatmap showing the expression level of the different DEGs identified in the Gene Ontology term G0:0042110 – T cell activation. **(F)** Graphical representation of the top 15 enriched terms on Biological Process based on the GSEA analysis on early deceased patients and D5+ survivors at TP1. Positive NES associate with early deceased patients, negative NES associate with D5+ survivors at TP1. **(G)** Graphical representation of the top 15 enriched terms on Reactome based on the GSEA analysis on early deceased patients and D5+ survivors at TP1. Positive NES associate with early deceased patients, negative NES associate with D5+ survivors at TP1.

To determine the changes in the transcriptome of PMNs during septic shock, we performed bulk RNAseq of PMNs isolated from 11 patients affected by septic shock at two different time points: within 12 hours (TP1) and 5 days (TP2) from ICU admission. Samples at TP1 were further distinguished based on mortality: early deceased patients and patients who survived for at least 5 days (D5+ survivors) (**Figure 2A**).

When we compared early deceased patients with D5+ survivors within TP1, we found 177 differentially expressed genes (DEGs), of which 23 were associated with early deceased patients and 154 were associated with survivors (**Figure 2B**). On the contrary, when comparing survivors at TP2 with the same patients at TP1, we found only 15 DEGs, of which 2 were associated with TP1 and 13 with TP2 (**Figure 2C**). This suggests that changes in PMN transcriptomes are more likely to play a role in the survival of patients at the early stages of sepsis rather than in the resolution of the pathology.

To determine whether the transcriptomic signature found in early deceased patients or D5+ survivors was associated with the severity of the pathology, we correlated the normalized counts of each DEGs with the sequential organ failure assessment (SOFA) score assessed at TP1. We didn’t find any significant correlation; however, the expression of the lncRNA TNK-AS1 showed a negative correlation with the SOFA score close to statistical significance (*P* = 0.0608, R = -0.5811) (**Supplementary Figures 1A, B**).

Gene Ontology (GO) analysis performed with the DEG sets revealed that PMNs from D5+ survivors are characterized by a T cell-like signature (**Figures 2D, E**). To rule out any possible contamination from T cells, we estimated the cellular composition of our bulk-RNA samples using the deconvolution tools MCPCounter and quanTIseq (17, 18). Both tools showed that our samples were almost exclusively composed of neutrophils with no significant contamination from T cells (**Supplementary Figure 1C**). As neutrophils are known to express T cell receptor (TCR)-like immune receptors (26), we analyzed the TCR repertoire of these samples. Concordantly, with the increased expression of *CD3D*, *CD3E*, and *CD3G* in PMNs from D5+ survivors (**Figure 2E**), these cells also showed a higher (although not significant) number of unique TCR clonotypes (**Supplementary Figure 1D**). Further, Gene Set Enrichment Analyses (GSEA) confirmed that PMNs from D5+ survivors at TP1 show higher expression of genes involved in T cell activation (**Figure 2F** and **Supplementary Table 2**, negative Normalized Enrichment Score – NES). GSEA also showed that, on the contrary, PMNs from early deceased patients showed increased IFN-, IL-1- and NF-κB-signaling compared to D5+ survivors (**Figure 2G** and **Supplementary Table 3**, positive NES). This suggests that PMNs from early deceased patients might have a hyper-activated phenotype resulting from an increased type-I IFN and IL-1-family signaling. Finally, GSEA showed that PMNs from early deceased patients are characterized by the heightened expression of genes involved in the control of the cell cycle (**Figures 2F, G**).

Taken together, these results suggest that PMN hyper-activation during the initial stages of sepsis might be a key process leading to early death from septic shock.

#### PMNs From Early Deceased Patients Retain a Mitotic Signature

**Figure 3:**
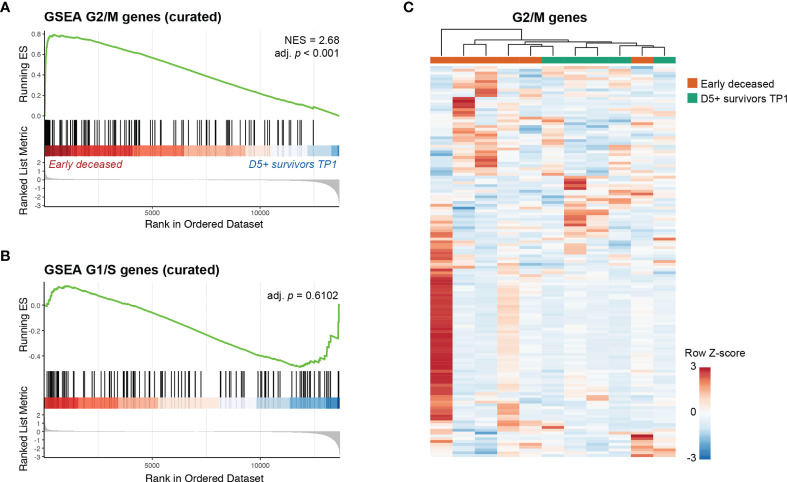
PMNs from early deceased patients show a transcriptomic signature associated with the G2/M cell cycle phase. **(A)** GSEA result obtained with the curated gene list of high confidence G2/M genes. **(B)** GSEA result obtained with the curated gene list of high confidence G1/S genes. **(C)** Heatmap showing the expression level of each gene in the curated G2/M dataset, for early deceased patients and D5+ survivors at TP1.

As GSEA analyses showed that PMNs from early deceased patients are characterized by a higher expression of genes involved in several cell cycle processes, such as “Regulation of chromosome segregation” and “Cyclin A/B1/B2 associated events during G2/M transition” (**Figures 2G, F**), we ought to further dissect the involvement of cell cycle genes. To understand whether this gene signature was associated with specific phases of the cell cycle, we utilized the results of a meta-analysis published by Fisher et al. (20) aimed to identify high confidence cell cycle-regulated genes. The analysis identified 258 genes associated with either the G1/S phase (118 genes) or the G2/M phase (140 genes) (**Supplementary Table 1**). GSEA analyses performed using this curated gene list, demonstrated that PMNs from early deceased patients are enriched in transcript involved in the G2/M phase (**Figure 3A**), but not in the G1/S phase (**Figure 3B**). No significant enrichments were found when comparing D5+ survivors at TP1 and TP2. **Figure 3C** shows the heatmap of the normalized read count for each G2/M gene.

Taken together, these results indicate that, compared to D5+ survivors, PMNs from early deceased patients retain a transcriptomic signature typical of the G2/M cell cycle phase suggesting a recent exit from the cell cycle.

### Proteome Analysis

**Figure 4:**
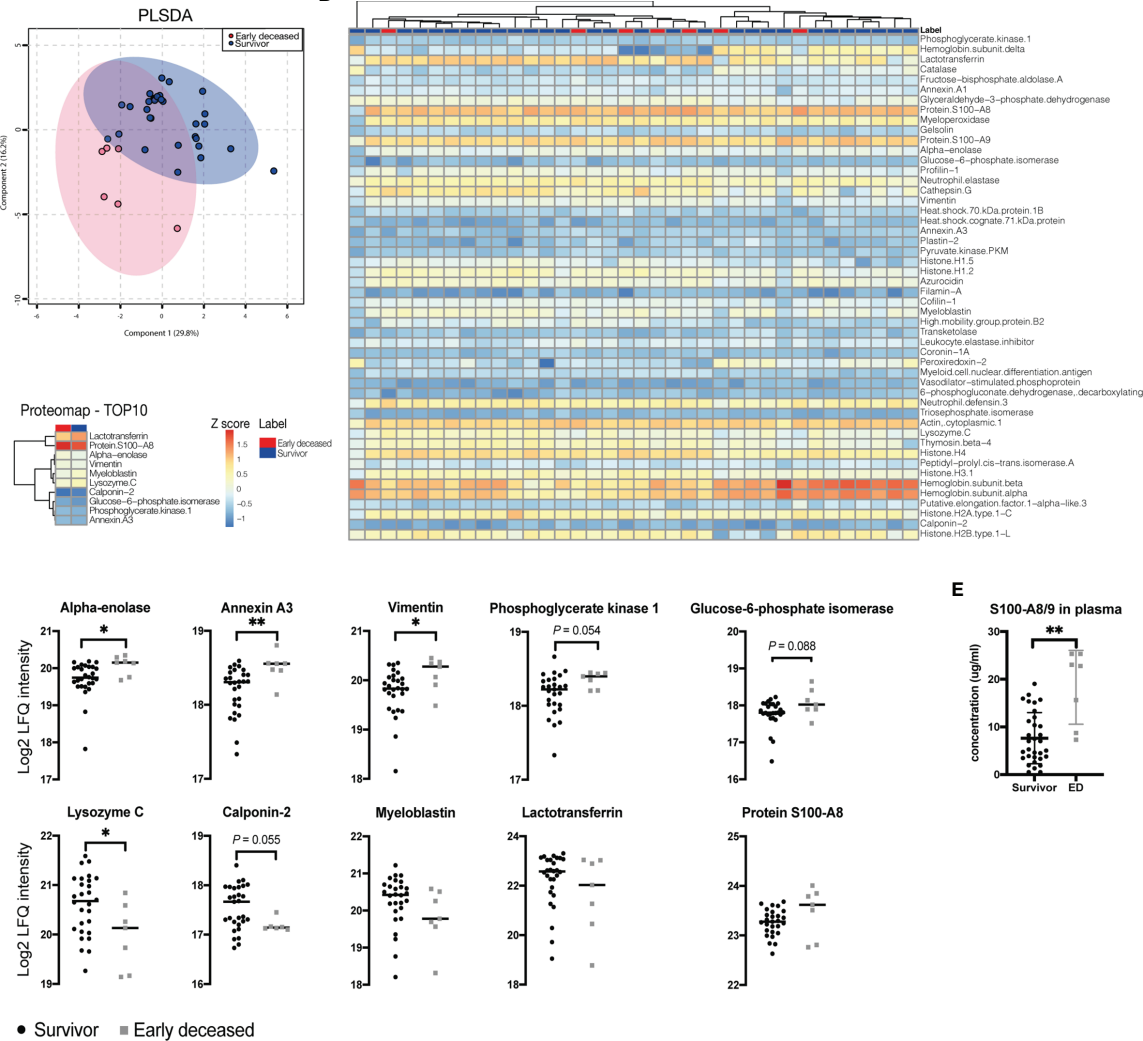
Overview and analysis of proteomics data. **(A)** Partial least squares discriminant analysis (PLSDA) revealed the difference between the groups of Early deceased patients and day 5+ survivors. **(B)** Heatmap showing the global expression of all the detected proteins. **(C)** Heat map showing 10 of the most differentially expressed proteins based on *P* value. **(D)** The LFQ intensities of the top 10 proteins were quantified and showed significant changes in alpha-enolase, Annexin A3, Vimentin, and Lysozyme **(E)** Plasma levels of alarmins S100A8/9 measured by ELISA. Data were tested with the Mann-Whitney test, error bars show SD. *(*P <*0.05), **(*P <*0.01).

By mass spectrometry, we revealed the overall proteomic profile of PMNs isolated from patients affected by septic shock. Partial least squares discrimination analysis showed the main differences between early deceased patients and D5+ survivors (**Figure 4A**). Globally, our analyses allowed us to detect 50 high-confidence proteins. The global expression of all measured proteins is visualized on the general heatmap (**Figure 4B**). We then analyzed the most differentially expressed proteins between early deceased patients and D5+ survivors (**Figures 4C, D**). We identified only 4 significantly differentially expressed proteins between the two groups (*P* value ≤ 0.05), specifically early deceased patients showed significantly increased levels of alpha-enolase, annexin A3, and vimentin (**Figure 4D**). The levels of phosphoglycerate kinase and glucose-6-phosphate isomerase showed a similar trend, but the difference was only close to a significant level. On the other hand, the patients that survived for more than 5 days showed a significantly higher amount of lysozyme C and the expression of calponin 2 showed a similar trend. Our data also suggest that myeloblastin and lactotransferrin levels were increased in D5+ survivors, but the difference didn’t reach statistical significance. Furthermore, we observed that the levels of S100A8 differ between D5+ survivors in comparison to early deceased patients at a proteomic level which is consistent with previously published studies (27). To confirm it, we analyzed plasma levels of S100A8/9 by ELISA and we found that early deceased patients showed significantly higher plasma levels of S100A8/9 (**Figure 4E**).

**Figure 5:**
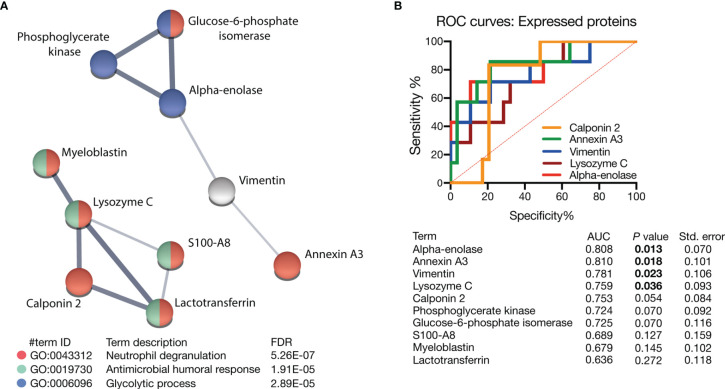
Biological functions and biomarker analysis of the most expressed proteins. **(A)** Proteins were analyzed by the STRING web tool to reveal their possible interactions and their association with specific biological processes. The line thickness between each node indicates the strength of the data support. **(B)** ROC curve analysis was performed to evaluate the potential to predict five-day survival. Alpha-enolase, annexin A3, vimentin, and lysozyme C showed the significant predictive potential of early death from septic shock.

**Figure 6:**
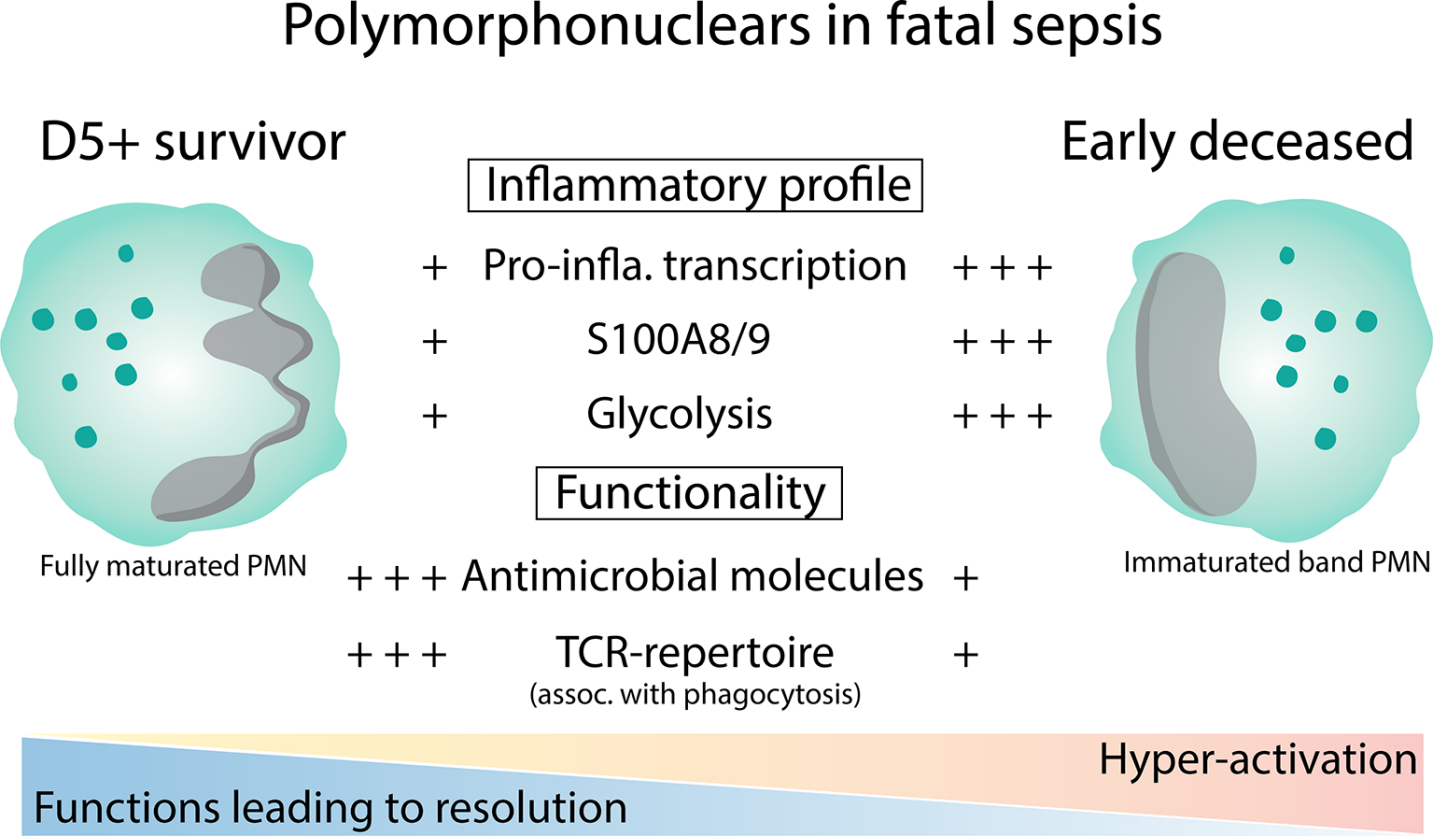
Polymorphonuclear cells show an altered activation status during fatal sepsis.

The ten most differentially expressed proteins were further analyzed using the STRING web tool and used to characterize possible protein-protein interactions (**Figure 5A**). Alpha-enolase, phosphoglycerate kinase, and glucose-6-phosphate isomerase were associated together with glycolytic processes, whereas glucose-6-phosphate isomerase, annexin A3, lactotransferrin, S100A8, calponin 2, lysozyme C and myeloblastin were linked to neutrophil degranulation. Myeloblastin, lysozyme C, S100A8, and lactotransferrin were associated with antimicrobial humoral response. Finally, we performed receiver-operator curve (ROC) analyses to evaluate the prognostic ability of these proteins. Alpha-enolase, annexin A3, vimentin, and lysozyme C all showed significant potential to predict short-term survival of patients affected by septic shock, with annexin A3 being the best prognostic marker (**Figure 5B**). Taken together, these data show that PMNs isolated from early deceased patients show traits of dysfunctional activation (**Figure 6**).

## Discussion

In this study, we analyzed the transcriptomic and proteomic signature of PMNs isolated from 11 and 34 patients, respectively, affected by septic shock. To determine the changes in PMN phenotype detectable at the time of admissions leading to early death in ICU, we divided our cohort into two groups: early deceased patients and patients that survived for more than 5 days. Furthermore, to shed light on the PMNs signatures associated with the progression of the pathology, we further analyzed the survivor group in two time-points: 12h (TP1) and 5 days from ICU admission.

RNA-seq analyses showed that, despite a considerable variability between samples, the major differences in the PMN’s transcriptomic signature were found between early deceased patients and day 5+ survivors at TP1. On the other hand, very few genes were found to be regulated between TP1 and TP2 at PMNs from survivors with no clear signature depicting the sepsis progression/resolution. This suggests that deep changes in PMN status are more likely to contribute to early death rather than in the progression of the pathology. This is in agreement with a study showing that patients deceased of septic shock within the first 5 days from ICU admission mostly died because of primary infection-related multiple organ failure, while later in-ICU deaths were mainly caused by end-of-life decisions or ICU-acquired infections (28).

GSEA analyses revealed that, compared to day 5+ survivors at TP1, PMNs from early deceased patients are characterized by an increased IL-1-family signaling, type-I IFN signaling, and NF-κB signaling, which might result in their hyper-activation. Consistently with this hypothesis, Majer and colleagues (29) showed that, in a murine model of fungal sepsis, type-I IFN signaling controlled the recruitment and activation of monocytes and neutrophils, resulting in hyper-inflammation and fatal kidney (29). Similarly, other studies showed that mice lacking type-I IFN receptor are more resistant to LPS- and TNFα-induced lethal shock (30, 31). Moreover, in our recent study on a similar cohort of sepsis patients, we showed that the plasma levels of IL-8, IL-18, and IL-33 – three cytokines belonging to the IL-1-family – were significantly higher in early deceased patients compared to day 5+ survivors (4). Consistently with the literature, our data indicate that excessive type-I IFN and IL-1 family stimulation on PMNs during sepsis might be a hallmark of early in-ICU deaths, and support the hypothesis that targeting type-I IFNs at the earliest time points of septic shock might improve patients’ survival (32).

On the opposite side, PMNs from day 5+ survivors in comparison to early deceased patients showed a transcriptomic profile similar to that of activated T cells. These cells showed an increased expression of all three CD3 chains and expressed a higher (although not statistically significant) number of unique TCR clonotypes. Only a small fraction of the clones identified in our dataset were annotated on a database of known TCR clones and were all directed against cytomegalovirus antigens. Different studies demonstrated that at least some populations of human neutrophils express a functional, TCR-based variable immunoreceptor as well as the RAG1/RAG2 recombinase complex (26, 33, 34). A recent study on patients affected by bacterial meningitis showed that, compared to circulating neutrophils, neutrophils in the cerebrospinal fluid rapidly initiate a V(D)J recombination, which leads to an increased receptor repertoire diversity. Furthermore, engagement of TCRαβ on neutrophils isolated from healthy donors enhanced their phagocytosis of anti-TCRαβ coated baits (33). Taken together, these results suggest that early deceased patients’ PMNs might show a reduced phagocytosis ability as a consequence of the diminished expression of TCRαβ chains and of different components of the TCR signaling complex.

Moreover, RNA-seq analyses showed that early deceased patients’ PMNs have a transcriptomic signature compatible with that of cells in the G2/M phase of the cells cycle. Although it is known that circulating neutrophils and other mature PMNs are post-mitotic, we believe that these cells might be retaining a G2/M-like transcriptional profile as a result of an increased proliferation of myelocytes and pre-neutrophils in the bone marrow (35). Consistently with this hypothesis, we and others have shown that sepsis patients exhibit a higher proportion of immature PMN-myeloid-derived suppressor cells compared to healthy donors (36, 37). Similarly, a recent study identified a population of proliferative neutrophil precursors in the spleen of healthy mice, suggesting that such progenitors can egress the bone marrow while retaining their ability to proliferate (38). Although immature neutrophils and progenitors are most likely to be found in the low-density fraction of peripheral blood immune cells, our data indicate that also more mature, high-density PMNs might retain a signature suggesting their recent exit from the cell cycle.

Consistent with our RNA-seq result, proteomic analyses of PMNs isolated from early deceased patients suggested that these cells might be functionally defective. In particular, we found a differential expression of proteins involved in neutrophil degranulation and antimicrobial responses. The decrease in lysozyme C, myeloblastin, and lactotransferrin levels is in agreement with the impairment in PMN phagocytosis suggested by our transcriptomic study. Interestingly, early deceased patients showed higher levels of 3 proteins involved in the glycolytic pathway: Alpha-enolase (ENO1), Phosphoglycerate kinase 1, and Glucose-6-phosphate isomerase. This suggests that PMNs from early deceased patients might be characterized by higher rates of glycolytic metabolism which also boost the activity of the pentose phosphate pathway (PPP). Interestingly, it was demonstrated that NET formation requires a metabolic shift to PPP (39). Moreover, excessive NET formation promotes inflammation and tissue damage in sepsis (40, 41). We speculate that the metabolic shift to the glycolytic pathway observed in early deceased patients might lead to an excessive formation of NETs with subsequent damage to the host organism. Furthermore, it has been shown that NETs formation increased ENO1 expression (42) leading to further amplification of this harmful effect, underlining the hyperactivation of PMNs.

PMNs from early deceased patients also showed decreased levels of anti-microbial defense proteins such as lysozyme C, calponin 2, and, to a lesser extent, myeloblastin and lactotransferrin. Except for calponin 2, these proteins are all abundantly present in human neutrophil granules and are important regulators of neutrophils’ antimicrobial activity (43). On the opposite side, we found higher levels of the alarmin S100A8/A9 in the plasma of early deceased patients compared to day 5+ survivors, and a similar trend was observed also at the proteomic levels in PMNs. S100A8/A9 are important immune-mediators that makeup approximately 45% of the cytoplasmic proteins in neutrophils (44). These proteins play a key role in the recruitment of leukocytes to the site of infection, promote the expression of several cytokines which sustain and exacerbate inflammation, and exert antimicrobial activity against a plethora of microorganisms (44). However, this complex has also anti-inflammatory properties as demonstrated in a murine model of acute asthma, where S100A8 suppressed mast cell degranulation and eosinophil infiltration in the lungs (45). Moreover, exogenous S100A8 administration reduced PMN infiltration and attenuated tissue damage in a murine model of LPS-induces endotoxemia (46). Consistent with the hypothesis of an excessive formation of NETs and impaired phagocytosis, these results indicate that PMNs from early deceased patients are characterized by a defective activation that might result in reduced pathogen clearance and direct damage of host organism.

Finally, we found that PMNs from early deceased patients expressed higher levels of annexin-A3 (ANXA3) and vimentin. Annexin-A3 is part of a family of calcium-binding proteins known to promote granule fusion in PMNs (47) and to increase neutrophil migration in hepatocellular carcinoma (48), while vimentin is an intermediate filament found in non-muscle cells (49). Interestingly, both proteins are known to affect cellular apoptosis by modulating caspase-3 activity. For example, the overexpression of vimentin in Jurkat cells reduced the apoptosis of cells after exposure to LPS (49). The authors also showed that, the expression of vimentin by lymphocytes was significantly higher in sepsis patients compared to healthy controls; furthermore, among sepsis patients, the vimentin level on lymphocytes was significantly higher in deceased patients (49). Moreover, a recent meta-analysis of public transcriptome data found that ANXA3 expression is almost completely restricted to neutrophils and that its gene expression level, as well as plasma levels, are increased in sepsis patients (50). Although the role of ANXA3 in increasing or dampening cellular apoptosis seems to be specific for different cell types, several studies showed that high ANXA3 levels are associated with reduced apoptosis (51–54). Moreover, it was recently described that in neutrophils, the cleavage of caspase-3 which, in turn, activates the caspase-3-mediated apoptotic pathway, is regulated by proteinase 3 (i.e. myeloblastin) (55). Interestingly, among the detected proteins, annexin A3 levels resulted to be the best predictor of early death for septic shock in ICU. Our results, thus, indicate that early deceased patients accumulate neutrophils with impaired functionality and prolonged lifespan.

Although our PMN purification approach and the limited number of patients analyzed didn’t allow us to draw a more definitive conclusion on specific cell types nor to better stratify our cohort, based on integrating gene expression and proteomic data, we showed that PMNs isolated from early deceased septic patients show signs of hyperactivation (possibly due to excessive type-I IFN signaling), impaired functionality, recent exit from the cell cycle, and prolonged life-span (suggested by annexin A3 and vimentin protein levels). Furthermore, we showed that annexin A3 levels on circulating neutrophils can be used as a predictor of early sepsis mortality in ICU. We believe that, albeit limited, our multi-omics approach highlighted some of the major PMNs’ dysfunctions during septic shock, and paved the road for future research aimed to identify valuable therapeutical targets in sepsis. We suppose that timely stratification of the group of vulnerable patients may improve their survival of septic shock by future personalized therapy targeting the altered polymorphonuclear functions.

## Data Availability Statement

The RNA-seq dataset is publicly available for download with the GEO accession number GSE180387. The proteome dataset is publicly available on the jPOST repository, with PXID - PXD029219.

## Ethics Statement

The studies involving human participants were reviewed and approved by St.Anne’s University Hospital, Institutional ethic committee, (4G/2018). The patients/participants provided their written informed consent to participate in this study.

## Author Contributions

MH-K designed the experiments, acquired the data, analyzed the data, and wrote the manuscript. MZ analyzed the data, wrote the manuscript, and secured funding. PL and KB designed the experiments and acquired the data. OM, IA, and AM participated in data collection. VŠ prepared the study protocol and the patient inclusion/exclusion strategy. MH prepared the study protocol and the patient inclusion/exclusion strategy, recruited the patients, and collected clinical data. VT recruited the patients and collected clinical data. JF conceptualized the study and secured funding. All authors contributed to the article, revised, and approved the submitted version.

## Funding

The research was supported by European Social Fund and European Regional Development Fund—Project MAGNET (No. CZ.02.1.01/0.0/0.0/15_003/0000492) and ENOCH (CZ.02.1.01/0.0/0.0/16_019/0000868), and by the Ministry of Health of the Czech Republic, grant nr. NU21-06-00408), all rights reserved and DRO (Institute of Hematology and Blood Transfusion – UHKT, 00023736). MZ was supported by the European Regional Development Fund - Project Support of MSCA IF fellowships at FNUSA-ICRC (No CZ.02.2.69/0.0/0.0/19_074/0016274). The CF Genomics of CEITEC was supported by the NCMG research infrastructure (LM2018132 funded by MEYS CR).

## Conflict of Interest

The authors declare that the research was conducted in the absence of any commercial or financial relationships that could be construed as a potential conflict of interest.

## Publisher’s Note

All claims expressed in this article are solely those of the authors and do not necessarily represent those of their affiliated organizations, or those of the publisher, the editors and the reviewers. Any product that may be evaluated in this article, or claim that may be made by its manufacturer, is not guaranteed or endorsed by the publisher.
