# Supplementary material for: Polymorphonuclear Cells Show Features of Dysfunctional Activation During Fatal Sepsis
Source: Front Immunol. 2021 Dec 13;12:741484. doi: 10.3389/fimmu.2021.741484 (PMC8710474; doi:10.3389/fimmu.2021.741484)
Supplement: Supplementary file 1 [file Image_1.pdf]

Polymorphonuclear cells show features of dysfunctional activation during fatal sepsis

Marcela Hortová-Kohoutková[1†], Marco De Zuani[1†], Petra Lázničková[1,2], Kamila Bendíčková[1], Ondřej Mrkva[1], Ivana Andrejčinová[1,2], Alexandra Mýtniková[1], Ondřej Polanský[1], Kamila Kočí[1], Veronika Tomášková[3], Vladimír Šrámek[3], Martin Helán[1,3], Jan Frič[1,4*]

[1] International Clinical Research Center, St. Anne's University Hospital, Brno, Czech Republic.

[2] Department of Biology, Faculty of Medicine, Masaryk University, Brno, Czech Republic

[3] Department of Anesthesiology and Intensive Care, Faculty of Medicine, Masaryk University, Brno, Czech Republic.

[4] Institute of Hematology and Blood Transfusion, Prague, Czech Republic

† These authors have contributed equally to this work and share first authorship

* Corresponding author: e-mail: jan.fric@fnusa.cz

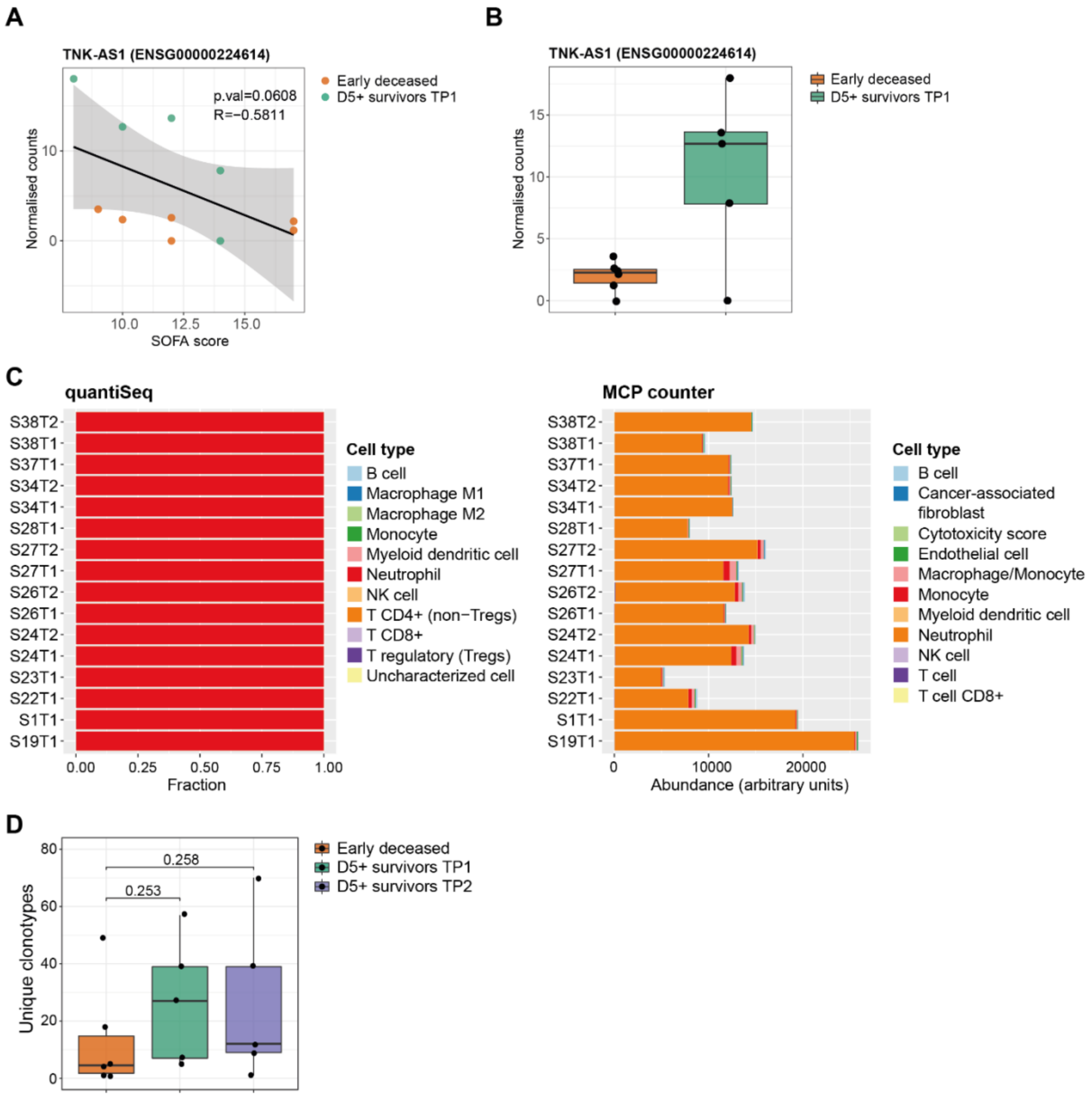

Supplementary Figure 1 | A) Plot describing the correlation between the normalized counts of *TNK-AS1* and the SOFA score of each patient. The black line indicates the linear regression and the grey area indicates its 95% CI. The numbers in the plot indicate the Spearman correlation coefficient and the *P* value of the correlation. **B)** Boxplot (Tukey) comparing the normalized counts of *TNK-AS1* in early deceased patients and D5+ survivors at TP1. **C)** Results of the seq deconvolution performed with quantiSeq and MCPCounter. **D)** Boxplot (Tukey) representing the number of unique TCR clonotypes in the three patient groups. Dots indicate the number of clonotypes identified in each sample. Pairwise comparisons were performed using Dunn’s test with Holm correction for multiple comparisons.
